# Broadly neutralizing antiviral responses induced by a single-molecule HPV vaccine based on thermostable thioredoxin-L2 multiepitope nanoparticles

**DOI:** 10.1038/s41598-017-18177-1

**Published:** 2017-12-21

**Authors:** Gloria Spagnoli, Somayeh Pouyanfard, Davide Cavazzini, Elena Canali, Stefano Maggi, Massimo Tommasino, Angelo Bolchi, Martin Müller, Simone Ottonello

**Affiliations:** 1 Department of Chemical Life Sciences & Environmental Sustainability, University of Parma, Parma, Italy; 2 Biopharmanet-Tec Laboratory, University of Parma and Interuniversity Consortium for Biotechnologies, Trieste, Italy; 3 German Cancer Research Center (DKFZ), Heidelberg, Germany; 4 International Agency for Research on Cancer, World Health Organization, Lyon, France

## Abstract

Vaccines targeting the human papillomavirus (HPV) minor capsid protein L2 are emerging as chemico-physically robust and broadly protective alternatives to the current HPV (L1-VLP) vaccines. We have previously developed a trivalent L2 vaccine prototype exploiting *Pyrococcus furiosus* thioredoxin (PfTrx) as a thermostable scaffold for the separate presentation of three distinct HPV L2(20–38) epitopes. With the aim of achieving a highly immunogenic, yet simpler and more GMP-production affordable formulation, we report here on a novel thermostable nanoparticle vaccine relying on genetic fusion of PfTrx-L2 with the heptamerizing coiled-coil polypeptide OVX313. A prototype HPV16 monoepitope version of this nanoparticle vaccine (PfTrx-L2-OVX313; median radius: 8.6 ± 1.0 nm) proved to be approximately 10-fold more immunogenic and with a strikingly enhanced cross-neutralization capacity compared to its monomeric counterpart. Vaccine-induced (cross-)neutralizing responses were further potentiated in a multiepitope derivative displaying eight different L2(20–38) epitopes, which elicited neutralizing antibodies against 10 different HPVs including three viral types not represented in the vaccine. Considering the prospective safety of the PfTrx scaffold and of the OVX313 heptamerization module, PfTrx-OVX313 nanoparticles lend themselves as robust L2-based immunogens with a high translational potential as a 3^rd^ generation HPV vaccine, but also as a novel and extremely versatile peptide-antigen presentation platform.

## Introduction

Different anogenital and other epithelial cancers are causally associated with infection by multiple human papillomavirus (HPV) types, fifteen of which are presently considered as oncogenic^1,2^. Although HPV 16 and 18 are responsible for approximately 70% of anogenital cancers, a significant fraction of this and other kinds of malignancies is caused by the remaining 13 oncogenic HPV types, whose prevalence can vary in different populations and geographic areas^3^. This multiplicity of high-risk HPV types somehow contrasts with the virus-specific immune responses induced by the current HPV vaccines (e.g., Gardasil® and Cervarix®), which are composed of virus-like particles (VLP) made of the type-specific major capsid protein L1 and elicit antibodies with very limited protection capacity against viral types not represented in the vaccine^1,4^. Furthermore, both L1-VLP vaccines suffer from a very limited thermal stability^5^, which hinders their use in low-resource countries, where HPV prophylaxis is most needed due to high infection rates and the lack of other preventive strategies.

These limitations have prompted great interest in HPV minor capsid protein L2 as an alternative antigen for the development of 3^rd^ generation, chemico-physically more robust prophylactic vaccines, capable of providing extended protection against cervical cancer and other HPV-related cancers as well as benign but clinically relevant lesions caused by multiple HPV types^4,6^. The need for a more broadly protective HPV vaccine is also attested by the recent release of a nonavalent L1-VLP vaccine (Gardasil® 9) affording protection against seven oncogenic HPV types plus two low-risk types (HPV 6 and 11)^7^. This is a significant advance compared to Gardasil® and Cervarix®, but coverage against the full set of oncogenic (and low-risk) HPV types is still far from complete. Moreover, the nonavalent vaccine, whose development has involved a considerable increase in formulation complexity and production costs, is also thermolabile and requires a continuous cold-chain distribution.

Because of the extensive sequence conservation of minor capsid protein L2 among different HPV types, L2-based antigens can confer a HPV protection much broader than that afforded by the L1-VLP vaccines^6^. However, linear L2-derived peptide epitopes, many of which have been mapped to a major cross-neutralizing epitope located in the N-terminal (aa. 17–38) region of the L2 protein^8,9^, are considerably less immunogenic than the conformational L1 epitopes displayed on full-length HPV L1-VLPs.

Different strategies aimed at overcoming this immunogenicity gap have been developed in recent years^6,9–17^. Some of these rely on particulate formulations such as the L1-VLP-L2 hybrid vaccine, which is built on a clinically validated but thermally unstable VLP scaffold^10,12,17^; the adeno-associated virus AAVLP-L2 vaccine^14,18^; the *Lactobacillus casei* HPV16-L2 vaccine^19^; and the bacteriophage MS2-VLP-L2 vaccine which has been shown to be extremely durable at room temperature when formulated as a spray-dried powder^20^.

A particularly robust L2-based antigen is a variant of our TDMI (Thioredoxin-Displayed Multipeptide Immunogen) L2 vaccine relying on thioredoxin from the hyperthermophilic archaeon *Pyrococcus furiosus* (PfTrx) as a macromolecular scaffold for the presentation of multiple copies (typically three tandem repeats) of HPV-L2 peptide (aa. 20–38) epitopes^21–23^. The PfTrx-L2 vaccine, whose production costs are extremely low, has been shown to be stable for 24 h at 100 °C and to withstand lyophilization as well as multiple freeze-thaw cycles. Its high conformational stability makes it also resistant to proteolysis and capable of accommodating polypeptide inserts longer than 200 amino acids^21^. Because of these favorable chemico-physical features, plus the lack of cross-reactivity of anti-PfTrx antibodies with human thioredoxin^21^, PfTrx lends itself as a very attractive scaffold for further engineering/optimization as a peptide epitope carrier and a promising candidate for clinical translation.

Two particular aspects of the PfTrx-L2 vaccine that remain amenable to further improvement are immunogenicity and the induction of further enhanced cross-neutralization responses. In fact, notwithstanding the generally broader protection afforded by L2-based immunogens, suboptimal protection against certain HPV types has been observed previously and dealt with through a trivalent mix formulation comprising L2 peptide epitopes derived from HPV31 and 51 in addition to HPV16^15,23^. Despite the increased protection broadness of this trivalent Trx-L2 vaccine^23^, a further desirable evolution would be its conversion into a single-molecule multiepitope form, allowing a more cost-effective and consistent GMP production in view of a phase I clinical trial.

Antigen immunogenicity has been shown to positively correlate with epitope multiplicity^9,24^, and a gain in immunogenicity -due to increased plasma half-life and lymph-node retention, plus an increased B-cell stimulation capacity- has previously been associated with an orderly increase in antigen size^25–27^. We thus decided to use a self-assembling polypeptide named OVX313, derived from the complement inhibitor C4-binding protein (C4bp)^28^, to convert our prototype monomeric Trx-L2 vaccine into a heptameric nanoparticle format. OVX313, which has been developed by OSIVAX and forms the basis of the OligoDOM® technology^29^, is a positively charged variant of an engineered C4bp derivative (IMX313) previously shown to lack cross-reactivity with its human counterpart and to increase the immunogenicity of (poly)peptide epitopes genetically fused to it^30–32^.

Following-up to the positive results of initial experiments carried out with PfTrx-L2 fused to OVX313, we redesigned the L2 epitope in order to include seven tandemly repeated peptide epitopes from a selected subset of high-risk HPV types, plus one L2 epitope from the low-risk but genital warts-causing HPV6 type. Cross-neutralization potency was further enhanced by this multiepitope single-molecule formulation (PfTrx-L2(8x)-OVX313), which elicited neutralizing antibodies against 10 different HPVs including three viral types not represented in the vaccine.

## Results

### Design and production of the PfTrx-L2-OVX313 antigen

**Figure 1:**
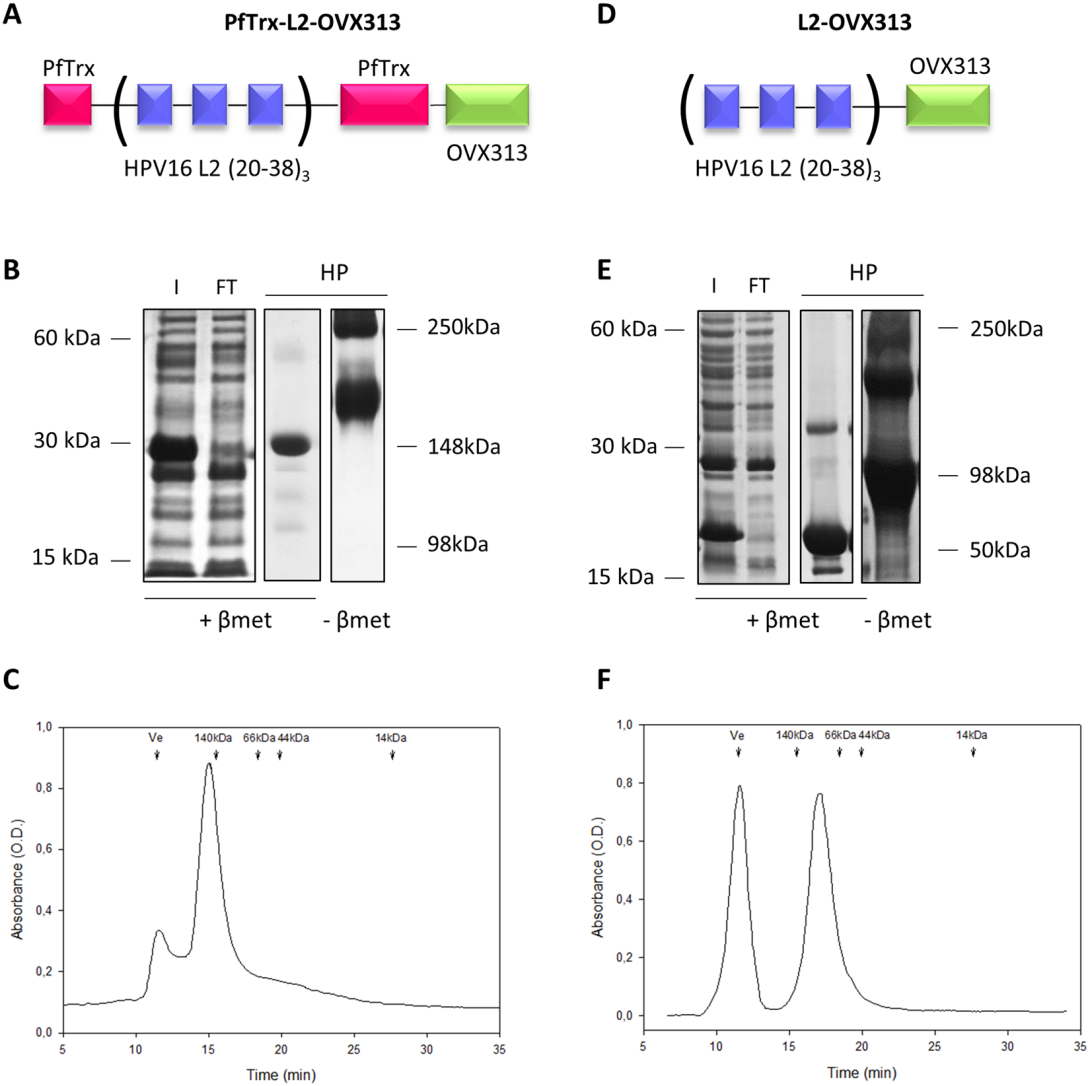
Design and production of the OVX313-containing L2 antigens. (**A**) Scheme of the PfTrx-L2-OVX313 construct. (**B**) SDS-PAGE analysis under reducing (+β-mercaptoethanol; +*βmet*) conditions of the total lysate from IPTG-induced *E. coli* cells overexpressing the PfTrx-L2-OVX313 antigen (*I*) and of the flow-through fraction (*FT*) obtained after heparin chromatography (see Fig. S2 for SDS-PAGE data on additional intermediate samples). The pool of salt-eluted, heparin-purified fractions (*HP*) electrophoresed under reducing (+*βmet*) or non-reducing (*−βmet*) conditions is shown in the last two lanes. Lanes *I* and *FT* were cropped from a single gel (see Fig. S7A for a full-length gel image); lanes labeled *HP* represent the results of single-sample SDS-PAGE analyses separately performed on the post-heparin pool. (**C**) Size-exclusion chromatography (SEC) analysis (see ‘Methods’ for details) carried out on heparin-purified PfTrx-L2-OVX313; Ve: excluded volume. The corresponding data for the PfTrx-lacking, L2-OVX313 construct are shown in panels D, E (see Fig. S7B for a full-length image of the gel from which lanes *I* and *FT* were extracted) and F, respectively.

A DNA fragment coding for the OVX313 polypeptide, made of 55 amino acids including two intermolecularly reactive Cys residues (see Fig. S1A), was genetically fused to a gene construct coding for our reference HPV16 monoepitope PfTrx-L2(20–38)_3_ antigen (hereafter designated as PfTrx-L2), which contains three tandem repeats of the L2 aa. 20–38 epitope separated by two Gly-Gly-Pro spacers in order to avoid junctional epitope formation^33,34^. A GlySer dipeptide was interposed between the N-terminus of the OVX313 oligomerization domain and the C-terminus of PfTrx-L2 in order to relieve a possible conformational interference between the two polypeptides in the fusion antigen^35,36^ (*PfTrx-L2-OVX313*; Fig. 1A). A similar strategy was applied to a HPV16-L2(20–38)_3_ construct directly fused to OVX313 without PfTrx (*L2-OVX313*; Fig. 1D), which served as a reference antigen to assess the contribution of PfTrx to L2 peptide immunogenicity in the context of the OVX313 heptamer. Both constructs were designed and produced as synthetic codon-optimized genes and transferred into the T7-RNA polymerase-dependent expression vector pET26b without the incorporation of any affinity-purification tag.

Following bacterial expression in a largely (>90%) soluble form (Fig. S2), the OVX313-fused L2 antigens were purified by two sequential chromatographic steps (see ‘Methods’ for details). The first purification step was conducted by adsorption to a heparin-affinity column, from which PfTrx-L2-OVX313 eluted at 0.3 M NaCl, while the L2-OVX313 protein (without Trx) eluted as a single peak at 0.8 M NaCl. As revealed by SDS-PAGE analysis carried out under conditions leading to complete reduction of the seven disulfide bonds that hold together the OVX313 heptamer, highly purified proteins with apparent molecular masses close to the expected ones (25 kDa and 13 kDa for PfTrx-L2-OVX313 and L2-OVX313, respectively) were recovered, with a total yield of approximately 15 mg of each antigen/liter of bacterial culture (Fig. 1B and E). Under non-reducing SDS-PAGE conditions, instead, two polypeptide bands were apparent: one corresponding to the expected size of the heptamers (~180 and ~90 kDa for PfTrx-L2-OVX313 and L2-OVX313, respectively) and a second, more slowly migrating band (close to the loading well) likely corresponding to a higher-order multimeric form of the proteins (~290 and ~180 kDa, respectively) (Fig. 1B and E; *lane* –*βmet*). This micro-heterogeneity, previously observed with other IMX313 constructs^31^, was confirmed by size-exclusion chromatography (SEC) analysis, which also yielded two peaks: a major one eluting with an apparent native molecular mass closely matching the size expected for the heptameric species and a minor peak eluting in the excluded volume, likely corresponding to a higher-order multimeric form of the protein (Fig. 1C and F). A similar partitioning, always with a marked prevalence of the heptameric form, was observed in different preparations, and comparable results in terms of purification and yield were obtained when the first heparin chromatography step was replaced by a cation exchange fractionation step (data not shown).

**Figure 2:**
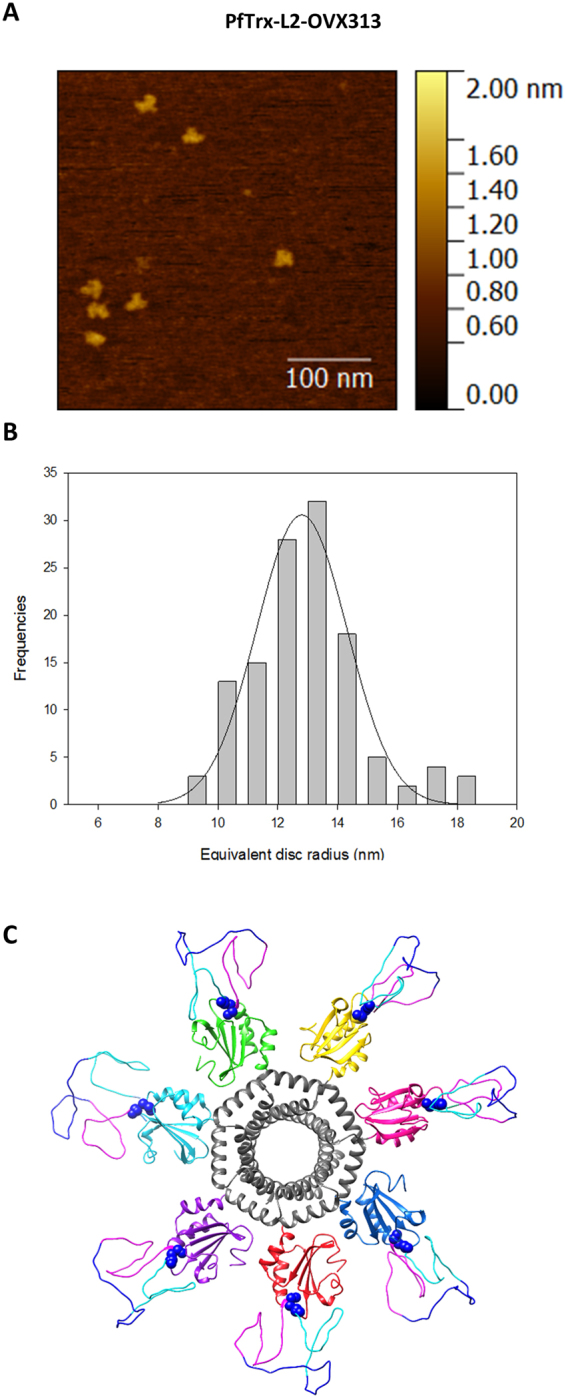
Atomic Force Microscopy analysis and structure prediction of the PfTrx-L2-OVX313 antigen. Representative topographic AFM image (**A**) and equivalent disc radius distribution (**B**) of the PfTrx-L2-OVX313 nanoparticles (see ‘Methods’ for details on AFM analysis and Fig. S3 for the construction of an equivalent radius calibration curve). (**C**) Predicted structure of the PfTrx-L2-OVX313 fusion protein generated with the SWISS-MODEL 3D structure prediction server and the MODELLER web service using *Gallus gallus* C4bp and an ancestral thioredoxin (PDB: 4ULX^38^) as templates.

More detailed insight on the assembly state of the PfTrx-L2-OVX313 nanoparticles was obtained by Atomic Force Microscopy (AFM), which as shown in Fig. 2 (panels A-B) revealed a fairly homogenous particle size distribution, with a median particle radius of 8.6 ± 1.0 nm (calculated with the use of bacterial RNA polymerase as a size standard^37^; see Suppl. Fig. S3). Interestingly, this AFM-derived size estimate is consistent with the size of the PfTrx-L2-OVX313 heptameric assembly predicted by a molecular modelling analysis conducted on the basis of the known three-dimensional structures of *Gallus gallus* C4bp (Lea *et al*. unpublished) and an ancestral thioredoxin (PDB: 4ULX^38^), which were used as structural templates for OVX313 and PfTrx, respectively.

Altogether, these results demonstrate the suitability of the reformulated PfTrx-L2-OVX313 antigen for high-level expression in *E. coli* as an autonomously assembled heptameric nanoparticle. Importantly, as revealed by circular dichroism (CD) analyses carried out at increasing temperatures (Fig. S4), the PfTrx-L2-OVX313 antigen, although less stable than PfTrx-L2^21^, can withstand at least a 10 min exposure to 80 °C without any apparent loss of secondary structure.

### Enhanced immunogenicity of the PfTrx-L2-OVX313 antigen

**Figure 3:**
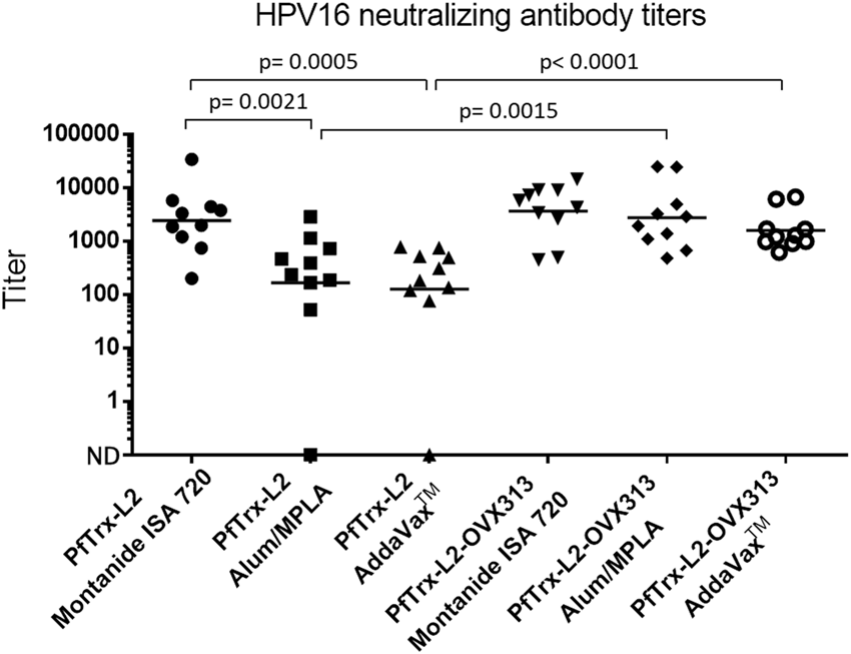
HPV16 neutralization titers elicited by the nanoparticulate PfTrx-L2-OVX313 antigen formulated with different immune-adjuvants. HPV16 L1-PBNA data obtained with immune-sera from mice (10 animals/group) immunized with the PfTrx-L2-OVX313 antigen formulated with the indicated adjuvants (see ‘Methods’ for details on the immunization protocol). The results of three parallel sets of immunizations performed with the reference PfTrx-L2 and with the nanoparticulate PfTrx-L2-OVX313 antigen are shown on the *left-hand* and the *right-hand* side respectively. Dots represent neutralization titers measured in individual vaccinated animals; the geometric means of the titers for each group are indicated by horizontal lines. Statistical significance (*p*-values) of the differences between the immune-responses measured in the different treatment groups is indicated.

An exploratory immunization experiment was conducted to assess the immunogenicity of PfTrx-L2-OVX313 supplemented with different immune-adjuvants, using PfTrx-L2 not fused to OVX313 as reference antigen. To this end, purified and detoxified antigens, formulated with either Montanide ISA 720, the human use-approved adjuvant Alum-MPLA, or AddaVax^TM^ (an analogue of the influenza vaccine-approved adjuvant MF59), were administered to mice (10 animals/group) using a one-priming, three-boosts immunization schedule, followed by determination of HPV16 neutralization titers measured with the highly stringent pseudovirion-based neutralization assay (L1-PBNA; see ‘Methods’ for details). As shown in Fig. 3, a significant immunogenicity enhancement (up to 10-fold) upon fusion of PfTrx-L2 to OVX313, was observed with Alum-MPLA and AddaVax^TM^, but not with the strong adjuvant Montanide ISA 720 (approved for therapeutic use only).

**Figure 4:**
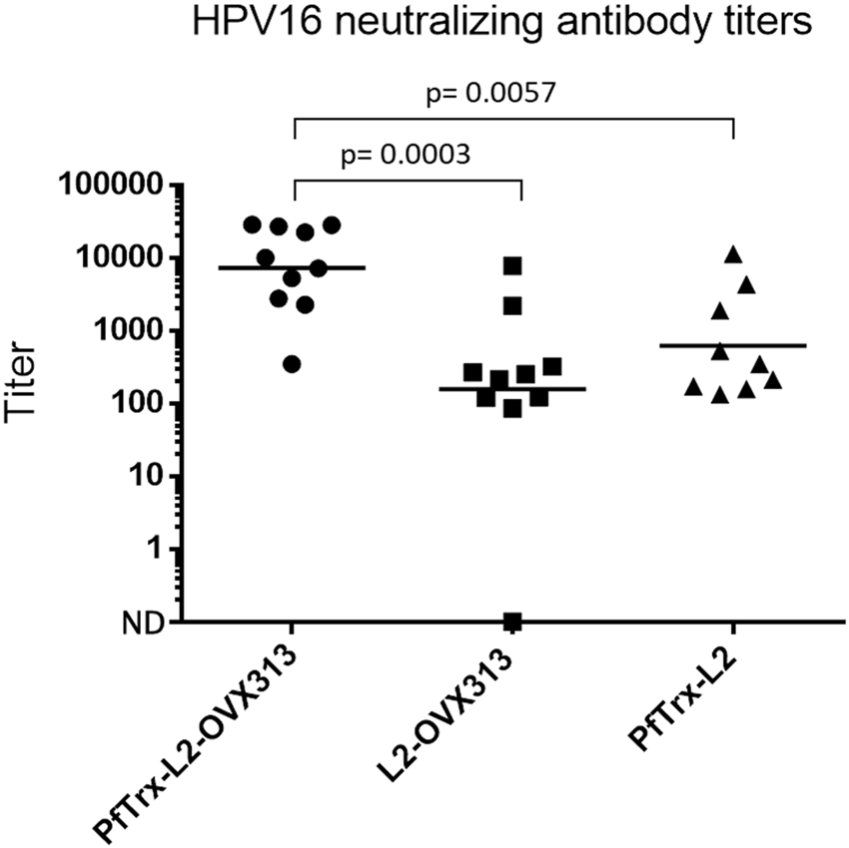
Neutralizing antibody induction capacity of different molecular formulations of the HPV16-L2(20–38)_3_ antigen. HPV16 L1-PBNA data obtained with immune-sera from mice (10 animals/group) immunized with the AddaVax^TM^- adjuvanted PfTrx-L2-OVX313, L2-OVX313 and PfTrx-L2 antigens, as indicated. HPV16 L1-PBNA data presentation and analysis are as specified in Fig. 3 legend.

We followed up to this result with a second immunization experiment aimed at estimating the relative contribution of PfTrx to the strength of the PfTrx-L2-OVX313 antigen by comparing the immunogenicities of PfTrx-L2-OVX313, PfTrx-L2 and L2-OVX313, all formulated with AddaVax^TM^. As shown in Fig. 4, fusion of PfTrx-L2 with OVX313 strikingly increased (>10-fold) anti-HPV16-L2 neutralization titers compared to the other two antigens. Although clearly less immunogenic than the double-fusion PfTrx-L2-OVX313 antigen, L2-OVX313 also elicited well detectable neutralization titers. The PfTrx-L2 antigen performed slightly better than L2-OVX313, although the difference was not statistically significant. This suggests a synergistic increase in immunogenicity brought about by OVX313-mediated heptamerization and thioredoxin-supported presentation of the HPV16 L2 tripeptide epitope.

Altogether, the above results indicate a significant immunogenicity enhancement brought about by the OVX313 oligomerization domain. This is only detectable in the presence of relatively mild, human use-approved adjuvants such as Alum-MPLA and AddaVax^TM^/MF59, both of which represent excellent candidates for clinical transfer studies. The practically unchanged immunogenicity observed with Montanide ISA 720, regardless of the presence of the OVX313 module, may reflect the superior immune-stimulatory strength of this reactogenic, therapeutic-use only adjuvant and/or the attainment of an immune-response saturation effect somehow related to the specific configuration of the thioredoxin-displayed L2 tripeptide epitope. Furthermore, only a two-fold enhancement of total anti-L2 titers, compared to the approximately 10-fold enhancement of HPV16 neutralization titers (Fig. 3), was revealed by a GST-L2-ELISA (not shown) comparing anti-L2 antibodies levels elicited by PfTrx-L2-OVX313 and the reference PfTrx-L2 antigen. This suggests that a predominantly qualitative (i.e., higher proportion of neutralizing antibodies), rather than a purely quantitative (i.e., general increase of anti-L2 antibodies) effect is associated to the immunogenicity enhancement brought about by the OVX313 heptamerization module.

### Conversion of PfTrx-L2-OVX313 into a single-molecule multiepitope antigen format

Another desirable feature of a clinically translatable 3^rd^ generation L2-HPV vaccine would be the achievement of broad-range protection with a thermally stable and low-cost single-molecule antigen easy to produce in GMP form. Building on our prior experience with the construction of large insert-containing Trx derivatives^9^ and on previous data pointing to the broadness of protection conferred by concatenated L2 peptides^13,39^, we set out to design an L2 multiepitope comprising tandemly repeated copies of the aa. 20–38 peptides from selected HPV types. To this end, we focused on a 12-amino acids L2 sub-region (aa. 20–31) previously shown to be recognized by cross-neutralizing anti-HPV monoclonal antibodies raised with the use of a Trx-L2(HPV16) antigen^40^.

**Figure 5:**
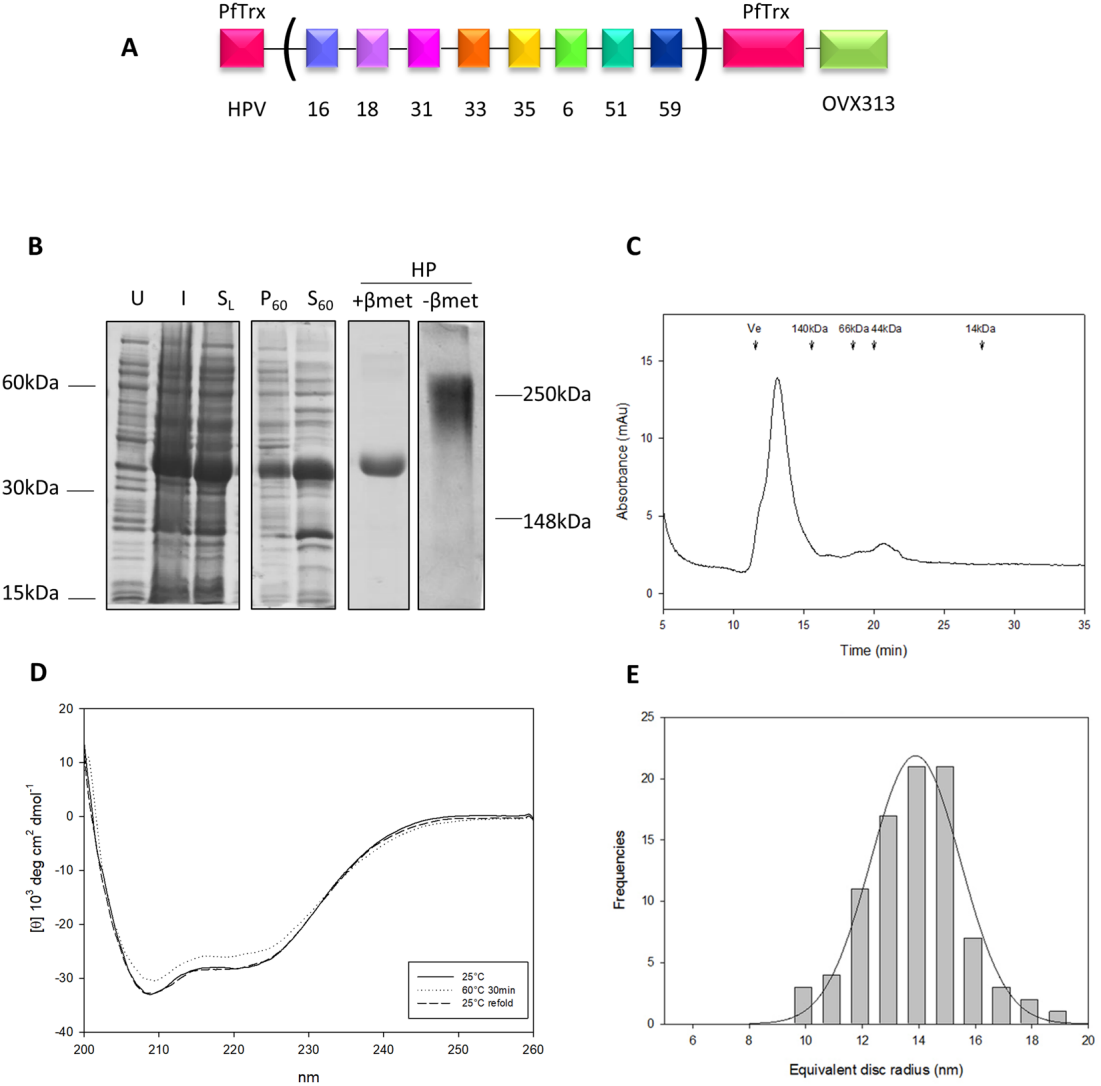
Design, purification and biochemical characterization of the multiepitope PfTrx-L2(8x)-OVX313 antigen. (**A**) Scheme of the PfTrx-L2(8x)-OVX313 construct (see Fig. S1 for the polypeptide sequence and Fig. S5 for the rationale underlying the choice of this particular set of L2 epitopes). (**B**) SDS-PAGE analysis under reducing conditions (+β-mercaptoethanol; *βmet*) of the total lysate from uninduced (*U*) and IPTG-induced (*I*) *E. coli* cells overexpressing the PfTrx-L2(8x)-OVX313 antigen, and of the resulting soluble lysate fraction (S_L_). The pelletable/heat-denatured (*P*_60_) and the soluble/heat-stable (*S*_60_) fractions recovered after heat-treatment of the soluble lysate for 30 min at 60 °C followed by centrifugation at 8,000 x g for 15 min are shown in the *fourth* and the *fifth* lane, respectively. The pool of salt-eluted, heparin-purified (*HP*) fractions electrophoresed under reducing (+*βmet*) or non-reducing (*−βmet*) conditions is shown in the last two lanes. Lanes *U, I* and *FT*, plus *P*_60_ and *S*_60_ were cropped from a single gel (see Fig. S7C for a full-length gel image); lanes labeled *HP* represent the results of single-sample SDS-PAGE analyses separately performed on the post-heparin pool. (**C**) Size exclusion chromatography analysis of the heparin-purified PfTrx-L2(8x)-OVX313 antigen; *Ve*, excluded volume (see ‘Methods’ for details). (**D**) Far-UV circular dichroism analysis of PfTrx-L2(8x)-OVX313 thermal stability carried out after heat-treatment at 60 °C for 30 min (*dashed line*) and after heat-treatment followed by a 10 min recovery at 25 °C (*dotted line*); the antigen kept at 25 °C served as an untreated sample control (*solid line*). (**E**) Equivalent disc radius distribution of the PfTrx-L2(8x)-OVX313 nanoparticles determined by AFM.

Following multiple alignment of the aa. 20–31 regions from 16 different oncogenic (α5, α6, α7, α9, α11) HPV types (Fig. S5), we identified eight sequence blocks corresponding to unique (HPV 31, 56 and 59) or shared (HPV 16 and 73; HPV 18, 68, 39 and 45; HPV 26 and 35; HPV 51 and 82; HPV 33, 52 and 58) L2 peptide epitopes, seven of which were selected as components of our multiepitope (“concatameric”) single-molecule antigen (Fig. 5A **;** but see also Fig. S1B). The HPV56 epitope, because of its previously observed inability to induce substantial neutralization titers when incorporated as a three-fold repeated independent unit into a HPV16-containing mix formulation (data not shown), was not included in the L2 multiepitope concatamer. The L2(20–38) peptide from the low-risk but highly prevalent HPV6 type, was added as the eighth epitope, despite its marked (89%) identity with the HPV 33 L2(20–38) peptide (Fig. S5 and data not shown). The combined L2(20–38) regions of each unique HPV type (31, 59 and 6) and of the most prevalent oncogenic HPVs (16, 18, 35, 51 and 33) in the case of multi-type groups, were converted into a codon-optimized DNA sequence (also encoding for inter-epitope GlyGlyPro spacers; see Fig. S1B), which was inserted into PfTrx and produced as a synthetic gene construct designated as PfTrx-L2(8x)-OVX313.

As shown in Fig. 5B, over 80% of the PfTrx-L2(8x)-OVX313 protein was recovered in the soluble, post-lysis supernatant fraction. Based on the previously observed thermal stability of the monoepitope PfTrx-L2-OVX313 antigen, this was subjected to an initial purification step carried out by heat treatment (30 min at 60 °C). Final purification was achieved by heparin-affinity chromatography (Fig. 5B, *lane HP*), followed by verification of heptameric assembly via non-reducing SDS-PAGE (Fig. 5B, *lane -β*-*met*) and SEC analysis (Fig. 5C) (see ‘Methods’ for details). As revealed by CD analysis, also the multiepitope PfTrx-L2(8x)-OVX313 antigen appears to be thermally stable and withstands the heat-treatment conditions utilized for thermal pre-purification without any apparent loss of secondary structure (Fig. 5D). Furthermore, as previously observed for lower multiplicity L2 inserts^41,42^, no more than 0.3 SH groups/molecule were found as free, 5,5-dithiobis-(2-nitrobenzoic) acid (DTNB)-reactive SH groups. This indicates a nearly complete oxidized state of, and disulfide bond formation by, the 140 cysteine residues present in the heptameric PfTrx-L2(8x)-OVX313 antigen (16 pairwise Cys residues associated to each L2 multiepitope, plus two Cys residues associated to each PfTrx and OVX313 module).

Also shown in Fig. 5 (panel E) is the size distribution of the PfTrx-L2(8x)-OVX313 particles, which qualitatively resembles the distribution obtained for the unheated HPV16 monoepitope antigen, but with a slightly higher median radius (9.6 ± 1.0 nm), likely reflecting the expanded size of the L2(8x) multiepitope.

### Virus neutralization responses induced by the PfTrx-L2(8x)-OVX313 antigen

**Figure 6:**
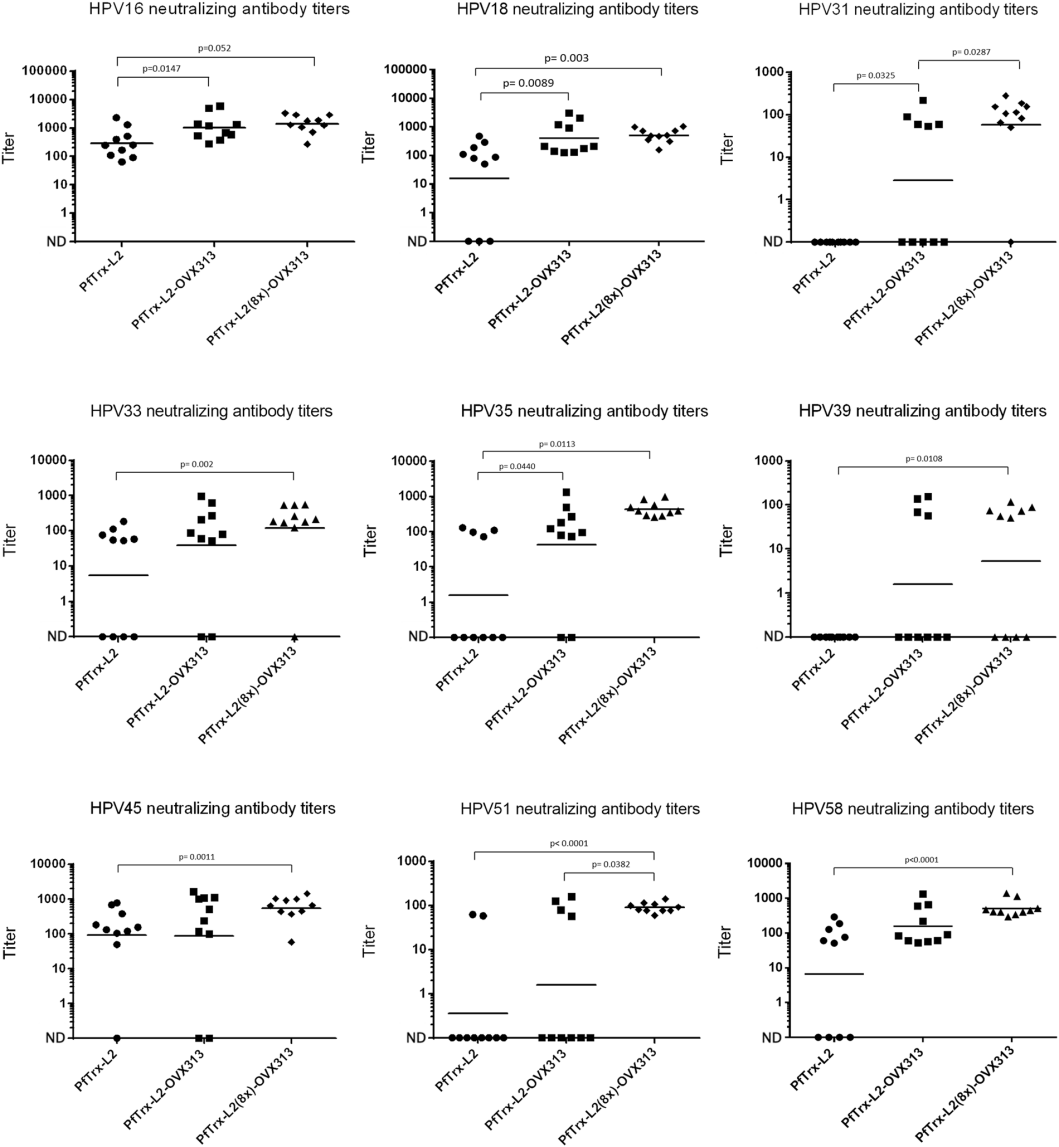
L1-PBNA neutralization titers against different HPV types elicited by immunization with the monoepitope and the multiepitope antigens. The results of L1-PBNAs conducted against the indicated HPV types with sera from mice (10 animals/group) immunized with the HPV 16 monoepitope (*PfTrx-L2-OVX313*) and the multiepitope (*PfTrx-L2(8x)-OVX313*) antigens adjuvanted with AddaVax^TM^ (50% v/v) are shown in each panel. L1-PBNA data presentation and analysis are as specified in Fig. 3 legend.

The impact of the multiepitope PfTrx-L2(8x)-OVX313 nanoparticle formulation on anti-HPV (cross-) neutralization responses was determined in a third set of immunization experiments, using as reference the HPV16 monoepitope PfTrx-L2-OVX313 and PfTrx-L2 antigens. To this end, the three antigens formulated with AddaVax^TM^ were administered intramuscularly to BALB/c mice (10 animals/group) using the same prime-boost regimen previously utilized for the PfTrx-L2-OVX313 antigen. Neutralization titers were determined against nine different HPV types, three of which (HPV39, 45 and 58) not included as type-specific epitopes in the PfTrx-L2(8x)-OVX313 antigen. As shown in Fig. 6, although neutralization titers varied from 1:1000 to approximately 1:100 with pseudovirions derived from different HPV types, the immunological performance of the OVX313-conjugated antigens was always higher and/or more consistent than that of the unconjugated PfTrx-L2 antigen, even in the case of HPV16 neutralization (Fig. 6, upper-left panel). Similar results, with a well detectable immune-response in all animals treated with the PfTrx-L2(8x)-OVX313 antigen, were obtained with the more sensitive L2 neutralization assay applied to HPV6 pseudovirions (data not shown).

A further enhanced neutralization broadness was elicited by the PfTrx-L2(8x)-OVX313 antigen, which outperformed the OVX313-containing HPV16 monoepitope antigen as well as the previous PfTrx-L2 trivalent formulation (data not shown), leading to a further potentiation of the overall strength and/or consistency of the immune response (Fig. 6; cf., for example, the responses elicited by the monoepitope and the multiepitope antigens against HPV 31, 51 and 35, and the non-vaccine types HPV 39 and 58).

We also tested the possible intrinsic adjuvancy provided by the OVX313 module. As revealed by experiments comparing the immunogenicity of PfTrx-L2(8x)-OVX313 with and without adjuvant, although some response was observed in all animals treated with unadjuvanted PfTrx-L2(8x)-OVX313, an over 10-fold reduction of neutralization titers and response consistency was observed with the unadjuvanted compared to the AddaVax^TM^-adjuvanted antigen (see Suppl. Fig. S6).

**Figure 7:**
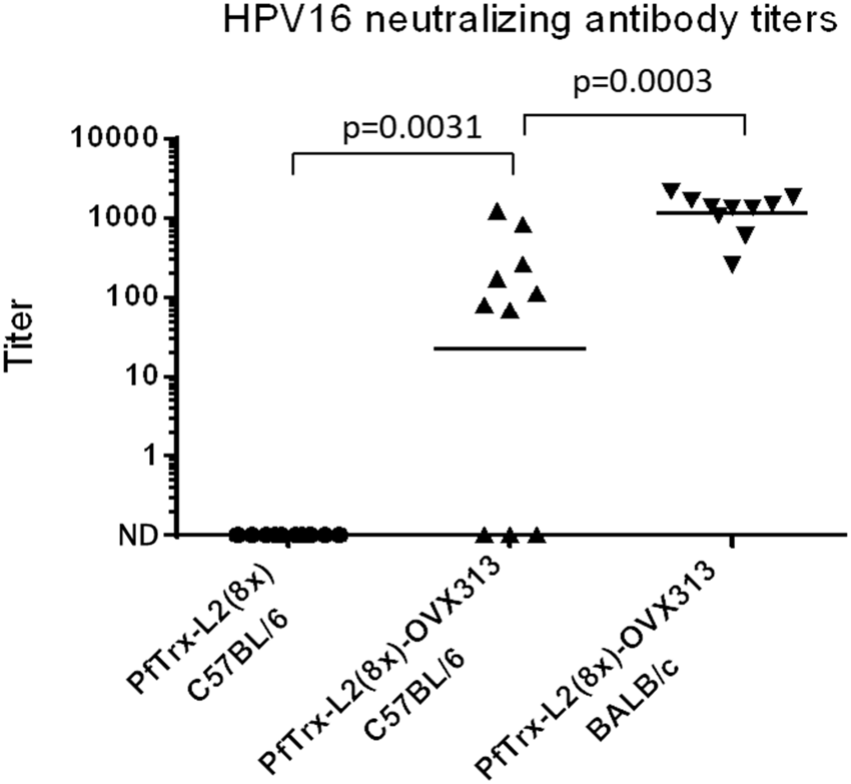
Immunogenicity of the PfTrx-L2(8x)-OVX313 antigen in C57BL/6 mice. AddaVax^TM^-adjuvanted PfTrx-L2(8x)-OVX313 was used for intramuscular immunization of H-2b (Th1-prevalent) C57BL/6 mice. The same mouse strain (C57BL/6) immunized with the reference PfTrx-L2 antigen and H-2d (Th2-prevalent) BALB/c mice immunized with PfTrx-L2(8x)-OVX313 served as controls. HPV16 L1-PBNA data presentation and analysis are as specified in Fig. 3 legend.

Knowing the relatively weak immunogenicity of the basic PfTrx-L2 antigen in C57BL/6 (H-2b, Th1 prevalent) compared to BALB/c (H-2d, Th2 prevalent) mice^14^, we also wished to investigate a possible contribution of the OVX313 super-scaffold to the anti-HPV immune response in this particular mouse strain. As shown in Fig. 7, a significant increase of anti-HPV16 neutralization titers was indeed observed in C57BL/6 mice immunized with the OVX313-conjugated multiepitope antigen, although the average response was not as high and consistent as that observed in BALB/c mice.

## Discussion

We have engineered and immunologically characterized a novel, nanoparticle-based system for (multi)peptide epitope presentation. This system relies on the combination, via genetic fusion, of two well established (poly)peptide antigen presentation technologies: thioredoxin-displayed multipeptide immunogens (TDMI)^9,21,22,43^ and C4bp-mediated immunogenicity enhancement via heptamerization^30–32^. Joint use of the two scaffolds increased immunogenicity by nearly the same extent compared to either single-scaffolded monoepitope antigen (PfTrx-L2 and L2-OVX313) when tested with the relatively mild, but human use-approved adjuvants Alum-MPLA and AddaVax^TM^ (equivalent to ASO4 and MF59, respectively). Although immunogenicity assessment was mainly based on *in vitro* L1-PBNA, it should be noted that this assay is by far the most stringent system for monitoring antibody-mediated prevention of HPV infection and that the results it produces are always confirmed (and magnified) by more sensitive assays such as the L2-PBNA and the *in vivo* passive transfer or challenge assays^23,44^.

Quite impressive, especially considering the weak adjuvanticity provided by OVX313, was the enhancement of cross-neutralization capacity induced by heptamerization. Multiple, non-mutually exclusive properties previously associated to larger size (20–200 nm) VLP (and VLP-like) antigens^23,25,27,45,46^, might be responsible for this potentiation effect. They include an increased drainage to lymph nodes, the supply of additional Th epitopes (as suggested by the OVX313-mediated increase in immunogenicity observed in C57BL/6 mice), plus an improved antigen internalization by APCs and presentation to T-helper cells. In fact, structured multimerization, as in VLPs and VLP-like particles, is thought to underlie the B-cell hyperstimulation observed with VLP-based and other particulate vaccines^26,27^. Another factor that might contribute to the potentiation effect elicited by the heptamerization module, is the presence at the OVX313 C-terminus of a cluster of basic amino acids (Fig. S1A). In fact, a net positive charge is known to promote intracellular internalization^47^ and the HIV-Tat Protein Transduction Domain (PTD) has recently been shown to enhance the humoral immunity and cross-protection induced by a recombinant HPV16 L2 peptide vaccine^48^.

Importantly, neutralization broadness, including HPV types not comprised in the vaccine, was consistently enhanced by conversion of the HPV16 monoepitope PfTrx-L2-OVX313 antigen into the multiepitope concatameric PfTrx-L2(8x)-OVX313 form. Although immune-response longevity data are not yet available, this single-molecule format, which is also amenable to DNA vaccination and viral-vectored antigen delivery, may turn out to be particularly useful for the streamlined and cost-effective GMP-production of a pan-HPV L2 vaccine.

Other particulate L2-based immunogens have been proposed in recent years as potentially effective 3^rd^ generation HPV vaccines^10,12,14,17–20^. The main advantages of the PfTrx-L2(8x)-OVX313 formulation are its ease and consistency of production as a soluble polypeptide in *E. coli* (a still preferred expression host for vaccine manufacturing^27^), the ability to induce broadly neutralizing responses, and its remarkable stability. The latter, which is expected to contribute to antigen persistence within the injection site, also relies on the covalent disulfide bond network that holds together the heptameric PfTrx-OVX313 nanoparticles. The above features, plus the lack of cross-reactivity of PfTrx with human thioredoxin^21^ and the positive safety profile obtained from a phase I trial of a candidate tuberculosis vaccine relying on an OVX313-related C4bp module^49^, point to the high clinical translation potential of the PfTrx-L2(8x)-OVX313 antigen as the active component of a novel low-cost, fully protective and chemico-physically robust HPV vaccine.

A final note regards the extreme versatility of the PfTrx-OVX313 system as a general-purpose macromolecular scaffold for conferring immunogenicity to any (poly)peptide epitope of interest. Indeed, ongoing work is focused on the exploitation of PfTrx-OVX313 for the construction of new multiepitope vaccines targeting α-type low-risk as well as cutaneous β-type HPVs, but also farm animals infecting PVs and other clinically relevant (including immunotherapeutic) targets.

## Methods

### Construct design and recombinant protein expression

Codon-optimized sequences coding for the PfTrx-L2-OVX313, L2-OVX313 and PfTrx-L2(8x)-OVX313 antigens, all bearing the OVX313 heptamerization domain sequence at the C-terminus, were chemically synthesized (Eurofins MWG Operon) and inserted into the *NdeI* site of a modified, tag-less pET26 plasmid (Novagen) (see Fig. S1). The resulting constructs were sequence-verified and transformed into *Escherichia coli* BL21 codon plus (DE3) cells for recombinant protein expression. Induction was performed by overnight culture (LB medium, 30 °C) in the presence of 1 mM isopropyl-β-D-thiogalactopyranoside (IPTG), followed by cell harvesting and washing, lysis via sonication, lysate centrifugation and soluble supernatant fraction recovery and processing as described previously^21,22^.

### Antigen purification

Heparin affinity chromatography (Hi Trap heparin columns, GE Healthcare) was used for one-step antigen purification. To this end, the supernatant fraction (typically 50 ml) derived from a 1-liter bacterial culture was loaded onto a Hi Trap column equilibrated in 25 mM Tris-HCl (pH 7.5), 150 mM NaCl. After washing with the same buffer (10 ml), elution was performed with a linear, 0.1 M-2 M NaCl gradient (30 ml) at a flow-rate of 1.0 ml/min using an ÄKTA Prime Plus (GE Healthcare) protein purification system. A pre-heparin, heat purification step (30 min at 60 °C, followed by centrifugation at 8,000 x g for 15 min to remove heat-labile precipitated proteins) was specifically applied to the PfTrx-L2(8x)-OVX313 antigen, which was then subjected to final purification (>95%) by heparin-affinity chromatography. Following SDS-PAGE analysis, individual peak fractions were pooled, exchanged into 25 mM Tris-HCl (pH 7.0), 300 mM NaCl buffer supplemented with the P8340 protease inhibitor cocktail (Sigma-Aldrich), and stored at either room temperature, 4 °C or −20 °C depending on the subsequent use of the proteins. 5,5-dithiobis-(2-nitrobenzoic acid) (DTNB) titrations were carried out on the purified PfTrx-L2(8x)-OVX313 antigen as described previously^41,42^. Antigens were detoxified by Triton X-114 (1% v/v) treatment^50^; endotoxin removal (to levels lower than 2 EU/ml) was verified in each sample by the LAL QLC-1000 test (Lonza).

### Gel filtration analysis

Size exclusion chromatography (SEC) was performed on a Superdex 200 HR10/30 column (24 ml; GE Healthcare) equilibrated in 25 mM Tris-HCl (pH 7.5), 150 mM NaCl, at a flow-rate of 0.7 ml/min, using an ÄKTA Prime Plus pump/monitoring (280 nm) system. A calibration curve was built with the use of yeast alcohol dehydrogenase (140 kDa), bovine serum albumin (66 kDa), ovalbumin (44.3 kDa), and lysozyme (14.5 kDa) as molecular mass standards.

### Far-UV circular dichroism

Far-UV CD spectra (200–260 nm) were acquired with a Jasco J715 Spectropolarimeter equipped with a Peltier temperature controller, using a 0.2 cm path-length cuvette, a bandwidth of 1 nm, a data pitch of 0.5 nm, and a response time of 4 s; CD spectra were averaged from 4 scans. Protein concentration was 7 µM in 10 mM phosphate buffer (pH 7.4). Following baseline correction, the measured ellipticity, h (mdeg), was converted to the molar mean residue ellipticity [Ѳ] (deg.cm^2^.dmol^−^), using [Ѳ] 5 h/cn_res_l, where Ѳ is ellipticity, c is the molar concentration of the protein, n_res_ is the number of amino acid residues in the protein and l is the optical path length in centimeters. Thermal unfolding studies were performed in the 25 °C–90 °C temperature range; protein samples were incubated for 10 min at different temperature (25, 40, 50, 60, 70, 80, 90 °C) before spectra analysis. For PfTrx-L2(8x)-OVX313, spectra were recorded at 25 °C, after incubation for 30 min at 60 °C, and at 25 °C after heat treatment in order to evaluate protein refolding.

### Atomic Force Microscopy

For AFM analysis, PfTrx-L2-OVX313 and PfTrx-L2(8x)-OVX313 nanoparticles and the bacterial RNA polymerase holoenzime standard (New England Biolabs) were diluted in deposition buffer (10 mM NaCl, 2 mM MgCl_2_, 4 mM HEPES, pH 7.4) at protein concentrations of 40, 45 and 80 nM, respectively. They were then deposited onto freshly cleaved mica for 1 minute, washed with milliQ water and dried with a gentile stream of nitrogen. AFM imaging was carried out in tapping mode in air with a Nanoscope IIIA (Digital Instruments) microscope equipped with an E scanner and an HQ:NSC14/Al BS tip (MikroMasch). Square images of 512_512 pixels were collected with a scan size of 1 μm. Images were analyzed with the Gwyddion software (v2.45) using the thresholding algorithm of the Gwyddion particle analysis procedure.

### Structure prediction

The SWISS-MODEL 3D structure prediction server^51^ was employed for structure prediction. The structure of monomeric PfTrx (GMQE score: 0.66) was built using as model the crystal structure of an ancestral thioredoxin (PDB: 4ULX) representing the last common ancestor of the Cyanobacterial, Deinococcus and Thermus groups^38^. The three-dimensional structure of *Gallus gallus* C4bp (Lea *et al*. unpublished) was used as template to build the structure of the heptameric OVX313 module. The MODELLER web service^52^ was used to predict and refine the putative structure of the three HPV-L2(20–38) epitopes.

### Immunization studies

Six- to eight-weeks-old female BALB/c mice (Charles River; Sulzfeld, Germany) were immunized four times at biweekly intervals with 20 μg of the various detoxified and filter-sterilized antigens. A constant weight amount of antigen, higher than the 15 μg dose of the basic PfTrxL2 antigen utilized in a previous study^23^ in which as little as 1 μg of antigen was found to be sufficient to elicit a maximal immune-response, was used to partly compensate for the difference in size between PfTrx-L2 (~25 kDa), PfTrx-L2-OVX313 (~180 kDa) and PfTrx-L2(8x)-OVX313 (~250 kDa). Antigens were adjuvanted with Montanide ISA 720 (Seppic, France; 50% v/v, subcutaneous injection), Alum/MPLA (Avanti Lipids; 50 μg and 5 μg, respectively; intramuscular injection) or AddaVax^TM^ (Invivogen; 50% v/v, intramuscular injection). Four weeks after the last immunization, blood samples were collected by cardiac puncture and sera were recovered after 2 hours at room temperature, by centrifugation at 4500 rpm for 10 min. Total anti-L2 antibody titers were determined by capture GST-L2-ELISA as described previously^9^.

### Neutralization assays

Pseudovirion preparation and L1-PBNAs were performed as previously described^41^. Briefly, 50 µl of serially diluted immune sera (or control monoclonal antibodies) were mixed with 50 µl of diluted PSV stocks and incubated at room temperature for 30 min. Next, 50 µl of HeLaT cells (2.5 × 10^5^ cells/ml) were added to the pseudovirion-antibody mixture and incubated for 48 h at 37 °C under a 5% CO_2_ atmosphere. The amount of secreted Gaussia luciferase was then determined in 10 µl of cell culture medium using the coelenterazine substrate and Gaussia glow juice (PJK) for detection as per manufacturer’s instructions. A microplate luminometer (Victor3, PerkinElmer) was used to measure luminescence in the culture medium 15 min after substrate addition. A previously described protocol was used for the L2-PBNAs^23,53^. Immune sera IC50s were determined using the GraphPad Prism program (GraphPad Software).

### Statistical analysis

The significance of neutralization assay results, and of the differences between different vaccine treatment groups, was determined with the non-parametric Mann-Whitney-Wilcoxon test performed with the GraphPad Prism software 5.00; differences between groups were considered significant at p < 0.05.

### Ethics approval

Animal experimentation procedures were approved by the Regierungspräsidium Karlsruhe under permit G19/13 and were performed in accordance with the relevant guidelines and regulations. Mice were kept and handled in the animal house facility of the German Cancer Research Center (DKFZ, Heidelberg) under pathogen-free conditions, in compliance with the regulations of the Germany Animal Protection Law.

### Data availability

All data generated or analyzed in this study are included in this published article and its supplementary information files. All methods were performed in accordance with the relevant guidelines and regulations.

## Electronic supplementary material

Supplementary Information
