# Supplementary material for: Broadly neutralizing antiviral responses induced by a single-molecule HPV vaccine based on thermostable thioredoxin-L2 multiepitope nanoparticles
Source: Sci Rep. 2017 Dec 21;7:18000. doi: 10.1038/s41598-017-18177-1 (PMC5740060; doi:10.1038/s41598-017-18177-1)
Supplement: Supplementary file 1 — Supplementary Information [file 41598_2017_18177_MOESM1_ESM.doc]

**SUPPLEMENTARY INFORMATION**

**Broadly neutralizing antiviral responses induced by a single-molecule HPV vaccine based on thermostable thioredoxin-L2 multiepitope nanoparticles**

Gloria Spagnoli^1^, Somayeh Pouyanfard^3^, Davide Cavazzini^1^, Elena Canali^1^, Stefano Maggi^1^, Massimo Tommasino^4^, Angelo Bolchi^1,2^, Martin Müller^3*^ and Simone Ottonello^1,2*^

^1^Department of Chemical Life Sciences & Environmental Sustainability, University of Parma,

Parma, Italy

^2^Biopharmanet-Tec Laboratory, University of Parma and Interuniversity Consortium for

Biotechnologies, Trieste, Italy

^3^German Cancer Research Center (DKFZ), Heidelberg, Germany

^4^International Agency for Research on Cancer, World Health Organization, Lyon, France

*Corresponding authors:

Simone Ottonello

Department of Chemical Life Sciences & Environmental Sustainability, University of Parma

Parco Area delle Scienze 23/A

43124 Parma, Italy

E-mail: s.ottonello@unipr.it

Martin Müller

German Cancer Research Center (DKFZ)

Im Neuenheimer Feld 280

69120 Heidelberg

Heidelberg, Germany

E-mail: martin.mueller@dkfz-heidelberg.de

**Supplementary Figures**

**Figure S1**

**
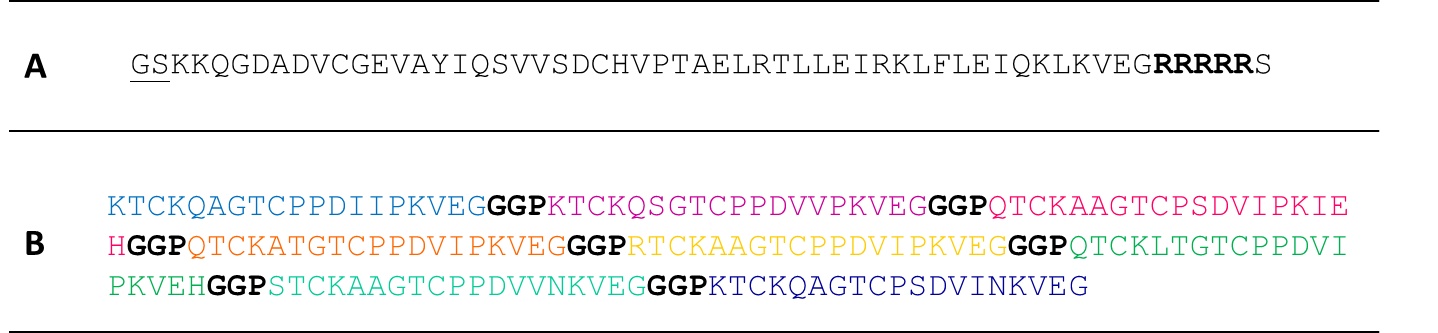
**

**Figure S1. OVX313 and L2(8x) polypeptide sequences.** (A) The GlySer dipeptide spacer interposed between the OVX313 N-terminus and the C-terminus of PfTrx-L2, and the five Arg residues present at the C-terminal end of the engineered OVX313 polypeptide are shown as *underlined* and *bold* one-letter code amino acid residues, respectively. (B) The polypeptide sequences of the eight L2(20-38) epitopes inserted into the PfTrxL2(8x)-OVX313 antigen (HPV 16, 18, 31, 33, 35, 6, 51, 59) are represented in different colors (same color-code as in Fig. 5); the tripeptide GlyGlyPro spacers interposed between individual L2(20-38) epitopes are shown in *bold*.

**Figure S2**

**
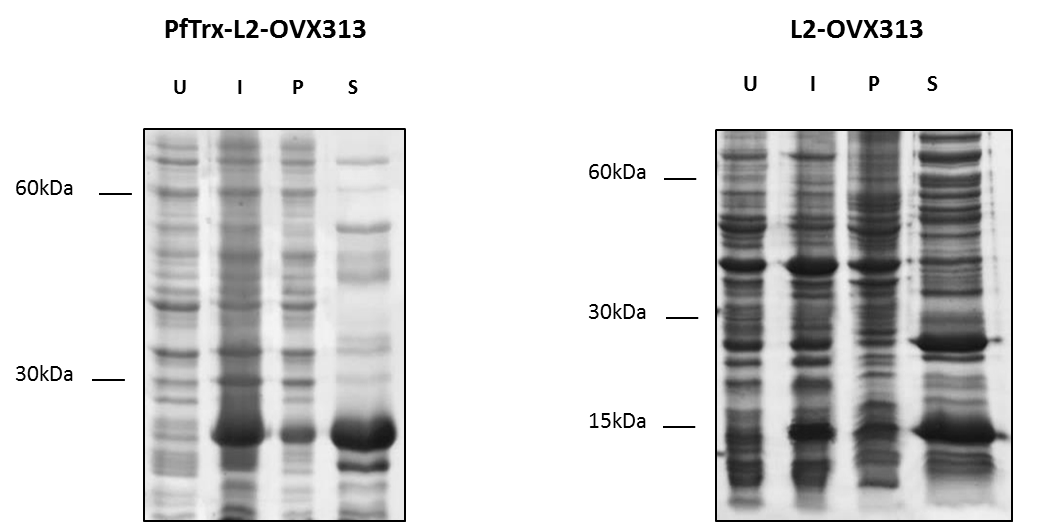
**

**Figure S2.** **Expression levels and solubility of the PfTrx-L2-OVX313 and L2-OVX313 antigens.** SDS-PAGE analysis of the total lysates from uninduced (*U*) and IPTG-induced (*I*) *E. coli* cells and of the pellet (*P*) and the soluble (*S*) fractions from bacterial cells expressing the PfTrx-L2-OVX313 (*left*) and the L2-OVX313 (*right*) antigens.

**Figure S3**

**
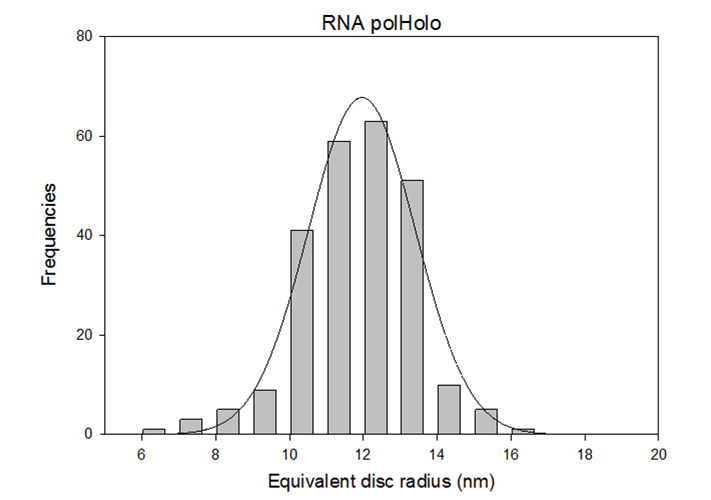
**

**Figure S3. Disc radius reference distribution utilized for AFM analysis**. Homogenously purified bacterial RNA polymerase holoenzyme (molecular mass: 460 kDa; major axis: 160 Å^1^) was utilized as a size standard for AFM grain analysis.

**Figure S4**

**
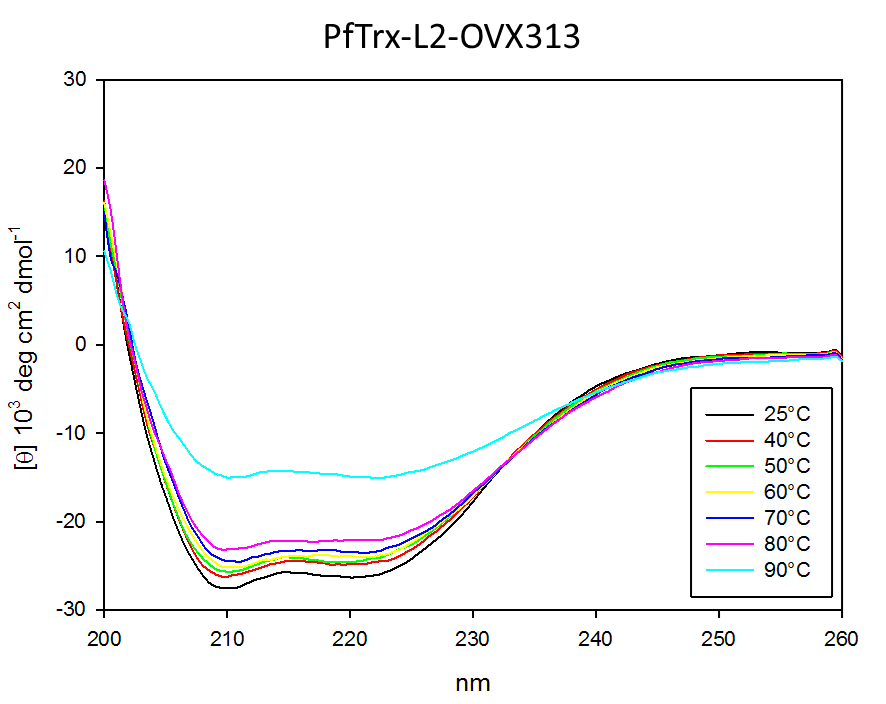
**

**Figure S4. Thermal denaturation of PfTrx L2-OVX313**. Far-UV CD spectra of PfTrx-L2-OVX313 were recorded after a 10 min heat-treatment at the indicated temperatures (25-90°C). Spectra are given as molar mean residue ellipticity [ϴ] (deg.cm^2^.dmol^-1^); [ϴ]=ϴ/cn_res_l, where ϴ is the ellipticity, c is the molar concentration of the protein, n_res_ is the number of amino acid residues, and l is the optical path length expressed in centimeters.

**Figure S5**

**
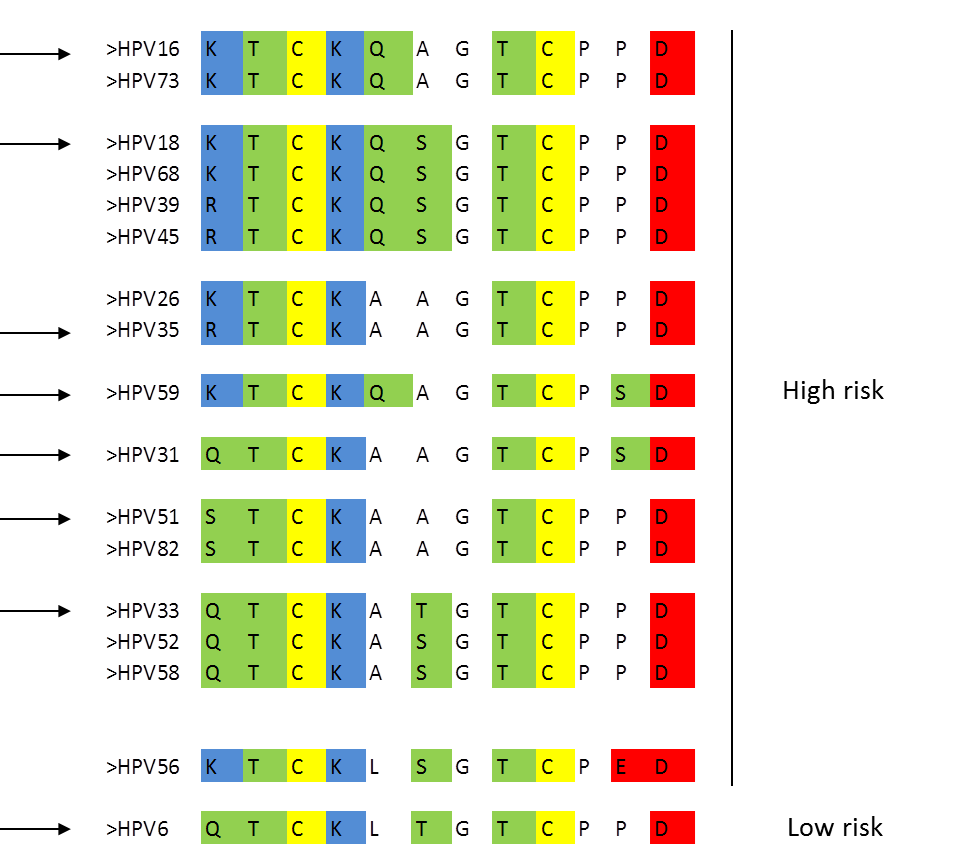
**

**Figure S5. Sequence alignment of the aa. 20-31 regions from high-risk HPVs and from the genital warts causing HPV 6 type.** Focusing on the aa. 20-31 sub-region recognized by cross-neutralizing anti-L2 monoclonal antibodies^2^, the indicated HPV types were divided into eight groups based on the amino acid chemical similarity observed within such region. *Green*: polar amino acids; *blue*: basic amino acids; *white*: non-polar amino acids; *red*: acidic amino acids; *yellow*: cysteine residues conserved in the L2 proteins from all HPV types. The eight HPV types whose aa. 20-38 peptides were included in the multiepitope PfTrx-L2(8x)-OVX313 antigen are marked with arrows.

**Figure S6**

**Figure S6. HPV16 neutralization titers elicited by the PfTrx-L2(8x)-OVX313 antigen formulated with and without adjuvant.** HPV16 L1-PBNA data obtained with immune-sera from mice (10 animals/group) immunized with the PfTrx-L2(8x)-OVX313 antigen formulated with AddaVaxTM or without adjuvant. Dots represent neutralization titers measured in individual immunized animals; the geometric means of the titers for each group are indicated by horizontal lines. Statistical significance (*p*-value) of the difference in the immune-responses measured in the two treatment groups is indicated.

**Figure S7**


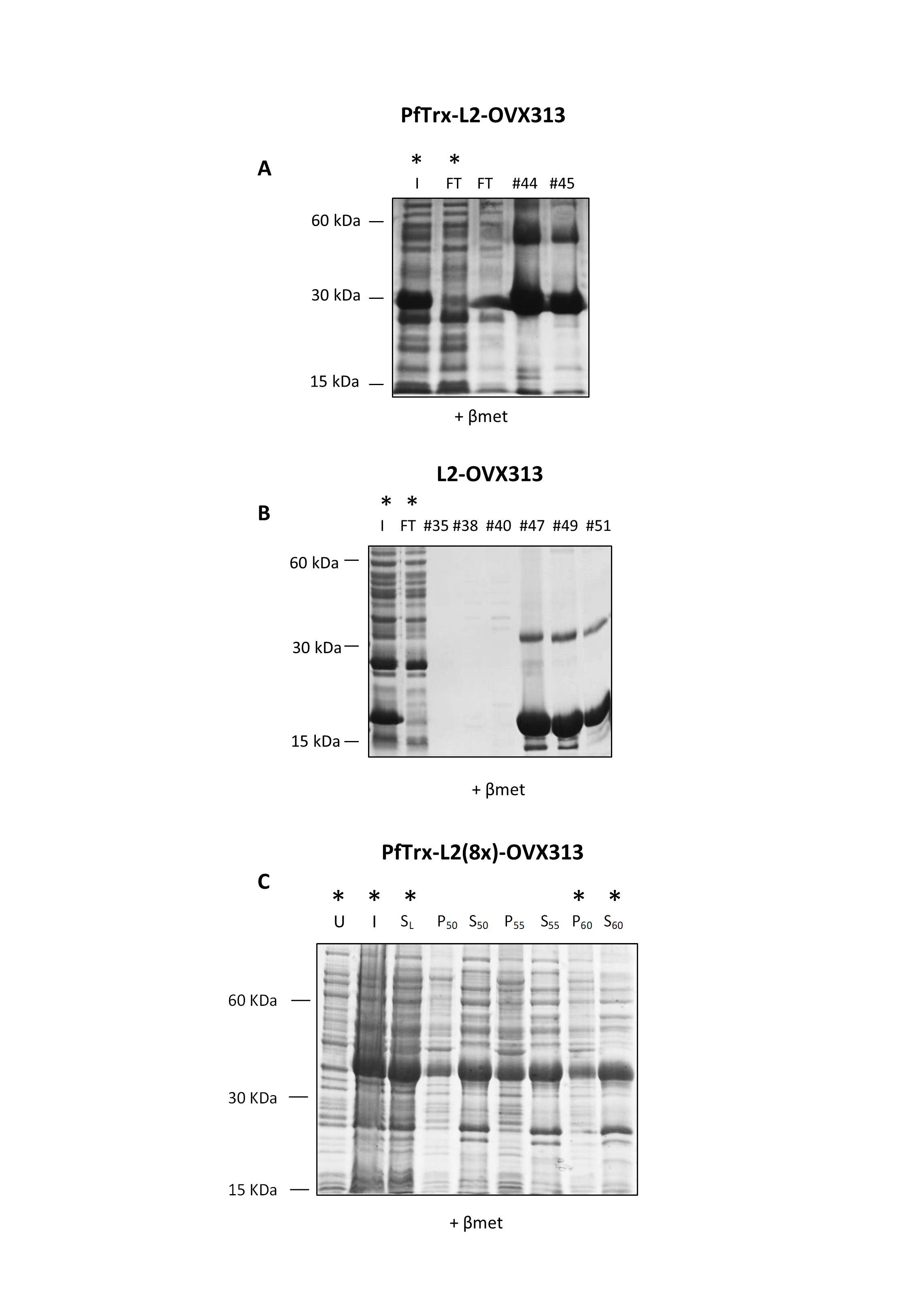


**Figure S7. Full-length SDS-PAGE data for intermediate fractions generated during PfTrx-L2-OVX313, L2-OVX313 and PfTrx-L2(8x)-OVX313 purification.** A) Total soluble lysate from IPTG-induced bacterial cells (*I*) overexpressing the PfTrx-L2-OVX313 antigen; heparin column flow-through (*FT*) fractions (different input amounts); and first two fractions (#44 and #45) eluted from the heparin column with a linear NaCl concentration gradient (see ‘Methods’ for details); another five fractions of increasing purity (not shown) were eluted with increasing NaCl concentrations and used to generate a pool of heparin-purified antigen fractions (*HP* in Fig. 1B). Asterisks indicate the two lanes that were cropped and shown in Fig. 1B (*leftmost-side*). B) Same as in (A) for the L2-OVX313 antigen; only one heparin flow-through (*FT*) fraction was loaded, followed by six fractions (#35 to #51) eluted from the heparin column with a linear NaCl concentration gradient; additional fractions (not shown) were eluted with further increasing NaCl concentrations and pooled (*HP* in Fig. 1E). Asterisks indicate the two lanes that were cropped and shown in Fig. 1E (*leftmost-side*). C) Uninduced (*U*), IPTG-induced (*I*) and soluble lysate (*S_L_*) fractions from bacterial cells overexpressing the PfTrx-L2(8x)-OVX313 antigen; these are followed by six lanes showing the pelletable (*P*) and the soluble/heat-stable (*S*) fractions recovered after heat-treatment of the soluble lysate for 30 min at the indicated temperatures (50°C, 55°C and 60°C) followed by centrifugation at 8,000 x g for 15 min. Asterisks indicate the lanes that were cropped and shown in the left-side of Fig. 5B (*U*, *I*, *S_L_*, *P_60_* and *S_60_*).

**References**

1. Finn, R. D., Orlova, E. V, Gowen, B., Buck, M. & Heel, M. Van. Escherichia coli RNA polymerase core and holoenzyme structures. **19,** 6833–6844 (2000).

2. Rubio, I. *et al.* The N-terminal region of the human papillomavirus L2 protein contains overlapping binding sites for neutralizing, cross-neutralizing and non-neutralizing antibodies. *Virology* **409,** 348–59 (2011).
